# Program directors’ perceptions of importance of pediatric procedural skills and resident preparedness

**DOI:** 10.1186/s13104-015-1499-8

**Published:** 2015-10-09

**Authors:** Zia Bismilla, Adam Dubrowski, Harish J. Amin

**Affiliations:** University of Toronto, 555 University Avenue, Toronto, ON M5G 1X8 Canada; Memorial University, St. John’s, Canada; University of Calgary, Calgary, Canada

**Keywords:** Medical education, Procedures, Residency, Pediatrics, Competency, Curriculum, Assessment, Simulation

## Abstract

**Background:**

The Royal College of Physicians and Surgeons of Canada (RCPSC) objectives for training in pediatrics include 26 procedural skills, 11 of which are included in the final in-training evaluation report (FITER). The importance of each procedure for practice and the preparedness of pediatric residency graduates to perform these procedures are not known.

**Methods:**

A questionnaire was distributed to all pediatric residency program directors and members of the RCPSC Specialty Committee in Pediatrics (N = 21) in October 2010, requesting them to rate the perceived importance and preparedness of graduating pediatric residents in all procedural skills on a 5 point Likert scale, as well as the presence of a curriculum and documentation for each procedure. Mean importance and preparedness were calculated for each procedure.

**Results:**

Response rate was 16/21 (76 %). Perceived preparedness was significantly lower than importance for the majority of procedures (p < 0.05). Ten procedures had a high mean importance rating (>3) but a low mean preparedness rating (<3). Presence of a curriculum and documentation for procedures varied across centers, and their presence was correlated with both perceived importance and preparedness (p < 0.0001).

**Conclusions:**

Many procedures in which pediatric residents are required to be competent by the RCPSC are felt to be important. Residents are not felt to be adequately prepared in several of the required procedures by the time of graduation. Procedures with high ratings of importance but low preparedness ratings should be targeted for curricular interventions.

**Electronic supplementary material:**

The online version of this article (doi:10.1186/s13104-015-1499-8) contains supplementary material, which is available to authorized users.

## Background

Procedural skills are an area identified as a training weakness in many countries and in many medical specialties [1, 2]. It has been well documented that pediatric residents have difficulty with acute care procedural skills and often do not complete the skills correctly [3–8].

### Curriculum and documentation

There are diverse ways in which programs may teach and document the procedural experience and skills of their residents. Trainees are typically taught and assessed through clinical preceptorships. Procedural skills that they may encounter in their day-to-day work during these preceptorships are observed by their clinical preceptors. These skills may be commented upon through an in-training evaluation report (ITER) at the end of each clinical rotation. In addition to day-to day clinical work, some programs have procedural skills curricula, which may include formal lectures, training from specialized teams [e.g. intravenous (IV) teams], hands-on training via animal models or simulation, or dedicated “procedure shifts” in environments where procedures tend to occur frequently (e.g. Emergency Department). Some programs have a log book (paper or electronic) in which residents record particular procedures they have performed on a rotation or throughout residency; some of these require the signature of an attending or supervising physician to corroborate the completion of the procedures, and others add an additional element of assessment of the performance of the procedure. Some programs formally assess procedural skills as part of an objective structured clinical exam (OSCE). For all Canadian programs, at the end of residency training, a final in-training evaluation report (FITER) is completed by the program director (PD), which includes 11 procedural skills that must be formally checked off to document procedural competency.

### Importance

A number of acute care skills have been described as essential for practice in a general pediatric setting [2]. The list of skills required of pediatric residents includes several skills in the area of acute care pediatrics, as well as in other areas such as administration of immunizations, curettage of the ear and bedside glucose measurement. These less acute skills have not been well studied, and their relative importance to pediatric practice is less well delineated.

### Preparedness

A study of US Pediatric program directors found that the list of procedures required by the US Residency Review Committee did not necessarily reflect the skills deemed most essential by PDs, that many residents were not prepared in several important procedures, and that trainees in programs where residents did not perform the majority of procedures were less likely to be judged competent in these procedures by graduation [2]. This study raised concerns that American residency programs may not be allowing a significant number of trainees to acquire skills necessary to practice in settings where the services available do not match those of their training center.

Similar concerns have been raised regarding Canadian pediatric residency programs [9–12]. The Canadian residency system and procedural training requirements in Canada are described in Box 1. Despite the list of procedures in the RCPSC objectives of training (OTR) in pediatrics, it is unclear which skills are specifically taught in Canadian pediatric residency program curricula, nor how experience in procedural skills is documented. It is not known what procedural skills are ultimately most important to pediatric practice; nor how competent residents are felt to be at these skills by the end of training. While some skills are included in the resident FITER, it is not known how the importance and preparedness for these particular skills compare to the skills not included in the FITER. This is becoming increasing important with the move towards competency based medical education and the focus on direct observation of skills for assessment [13–15]. The objectives of this study were to:

1. Determine the perceived importance of each of the pediatric procedural skills required by the RCPSC.
2. Determine the perceived preparedness of Canadian pediatric residents in the procedures required by the RCPSC.
3. Describe which procedural skills are formally taught and documented in Canadian pediatric residency curricula.

**Table:** **Box 1**

The Royal College of Physician and Surgeons of Canada (RCPSC) is the standard setting body for Postgraduate Medical Education in Canada
The RCPSC Specialty Committee in Pediatrics develops the objectives of training in pediatrics [16], which includes 26 procedural skills
To maintain accreditation, pediatric residency programs must include procedural skills curricula in their training programs
11/26 Procedural skills are specifically included in the Final In-Training Evaluation Report/Comprehensive Competency Report completed by pediatric program directors for each resident at the end of training
Core pediatric residency in Canada is a 4 year training program, with a comprehensive written and objective structured clinical exam (OSCE) at the end of training. Passing this exam confers eligibility to practice general pediatrics independently in Canada

## Methods

The Hospital for Sick Children Research Ethics Board provided approval for the study and participants were consented accordingly.

### Study design

#### Participants

Participants included all members of the Specialty Committee in Pediatrics (SCP) of the RCPSC. The SCP members are a select group of pediatricians from a range of practice settings (hospital, community, primary care, subspecialty, tertiary care etc.) involved in pediatric medical education. They are however not recent graduates. The SCP of the RCPSC is a standard setting body for residency training in pediatrics. The SCP develops the objectives of training, specialty training requirements, and the final in-training evaluation assessment tool including the contents of the FITER. Members represent PDs for each of the 17 pediatric residency programs in Canada, as well as a chair, vice-chair and members representing distinct geographic regions in Canada. There were a total of 21 members at the time of the study.

#### Questionnaire

A questionnaire including all procedural skills required of pediatric residents by the RCPSC (as contained in the RCPSC Pediatric OTR) was developed and modeled after a questionnaire by Gaies et al. [2] (see Box 2). Items on the questionnaire included all invasive and non-invasive procedural skills in the RCPSC pediatric OTR. This resulted in an initial list of 26 skills. Several skills were broken down into subcomponents: “cardiopulmonary resuscitation (CPR) (neonatal and pediatric)” was broken down into neonatal CPR, pediatric CPR and defibrillation; “Bag-mask ventilation (BMV) and tracheal intubation (neonatal and pediatric)” was broken down into (1) BMV, (2) neonatal tracheal intubation and (3) pediatric tracheal intubation; and “bladder catheterization and/or suprapubic aspiration” was broken down into (1) bladder catheterization and (2) suprapubic aspiration. This resulted in a total of 31 discrete skills for study. This questionnaire was pilot tested in a group of practicing general pediatricians and pediatric fellows at a tertiary care academic center, and modified based on feedback obtained during a focus group of these pediatricians. An electronic version of the final questionnaire was created.

**Box 2:** Model survey

Gaies et al. developed a survey based on the American Residency Review Committee’s (RRC) guidelines for procedural training. It included items about the importance of 29 procedures encountered in US pediatric training, estimates of residents’ preparedness in performing them, and the teaching of procedural skills. They collected (1) information about the perceived importance for residents to achieve preparedness in these procedures, rated on a 10 point Likert scale; (2) perception of resident preparedness to perform procedures; and (3) educational methods used by respondents for teaching procedural skills. With permission from Gaies et al., we developed our questionnaire using their survey as a template. We assessed the same 3 areas assessed by Gaies et al., and added documentation of skills as an additional item. We replaced the US RRC required procedures with the Canadian RCPSC pediatric procedures	

The first part of the questionnaire asked for demographics of the respondent including their status as a PD or other member of the SCP, year of completion of core pediatric residency training, and primary specialty or subspecialty. Respondents were then asked to describe characteristics of their pediatric residency program including the total number of pediatric residents in their program, the percentage of time their residents spend in hospital, community and ambulatory settings, the percentage of residents pursuing subspecialty training, and the percentage of residents pursuing subspecialty training in “proceduralist” fields (cardiology, critical care, emergency medicine and neonatology) as previously described by Gaies et al. [2]. Information about ancillary phlebotomy and IV teams and the frequency with which residents participate in these procedures was also collected. For each of the 31 skills included in the questionnaire, respondents were asked the following questions:

1. “In your opinion, how important is the competency of performing the following procedures?” Participants responded using a 5 point Likert scale with 1 being “not important,” 2 “somewhat important”, 3 “important”, 4 “very important” and 5 “extremely important”. Competency was defined as “the ability to perform a procedure or skill independently, without supervision, and with a high likelihood of successful completion. This includes an understanding of the indications, contraindications, and risks of the procedure or skill” [2].
2. “In your opinion, how well prepared are residents in your program to perform the following by the end of their residency?” Participants responded using a 5 point Likert scale with 1 being “not prepared”, 2 “somewhat prepared”, 3 “adequately prepared”, 4 “very well prepared”, and 5 “extremely well prepared”.
3. “Do you have a standardized curriculum for teaching this procedure or skill (yes or no)?” Curriculum was defined as any of the following: didactic sessions, formal observation of procedures performed by experts, or observed practice/simulation.
4. “Do you (the PD, or a delegate within the residency program) document the competence of your residents to perform this procedure or skill (yes or no)?” Documentation was defined as including procedure logs, evaluation using simulations, OSCE evaluation or other methods resulting in a formal record of preparedness.

In October 2010, an email was sent to each of the 21 members of the RCPSC SCP including a description of the study, an invitation to participate, and a link to the electronic questionnaire. The questionnaire was completed online, and was anonymous. Respondent emails were tracked by the questionnaire coordinator who was not a member of the research team. Participants that did not respond initially received a total of 3 reminder e-mails at 2-week intervals.

### Data analysis

Descriptive analyses of questionnaire responses was performed to characterize demographics of respondents and programs, the opinions surrounding the perceived importance of each procedure, estimates of the current level of perceived preparedness of pediatric residents at the respondents’ institutions, and curricula and documentation practices at pediatric training programs. We decided a priori to identify procedures with a mean rating ≥4 to be most important to pediatric practice. This is similar to the definition of importance used by Gaies et al. [2] as well previous similar studies [17, 18].

We likewise defined a priori a preparedness of ≥3 as adequately prepared and <3 not adequately prepared. Paired sample T tests were used to compare perceived importance and preparedness of each of the procedures.

Chi square and Fisher exact tests were used to assess whether perceived importance or estimated preparedness for a procedure was associated with respondent or program characteristics. Pearson’s correlations and regression analyses were used to analyze relationships between importance, preparedness, curricula and documentation. Two-sided p < 0.05 indicated statistical significance.

## Results

Response rate was 16/21 (76 %), with responses from 15 of the 17 Canadian pediatric residency program directors and the chair of the SCP. Five respondents described themselves are general pediatricians (3 “academic,” 1 “community,” and 1 “consulting”), and 11 as subspecialists (including neonatal-perinatal medicine, infectious diseases, critical care medicine, neurology, allergy/immunology, endocrinology/metabolism and adolescent medicine). Residents were noted to spend 77 % of their time in university hospital centers, 11 % in community hospital centers and 13 % in community ambulatory centers. Fifty percent of primary institutions had a 24-h phlebotomy team and 31 % had a daytime phlebotomy team. Program and respondent characteristics were not associated with perceived importance, preparedness, curriculum or documentation practices of various skills.

### Importance

**Fig. 1:**
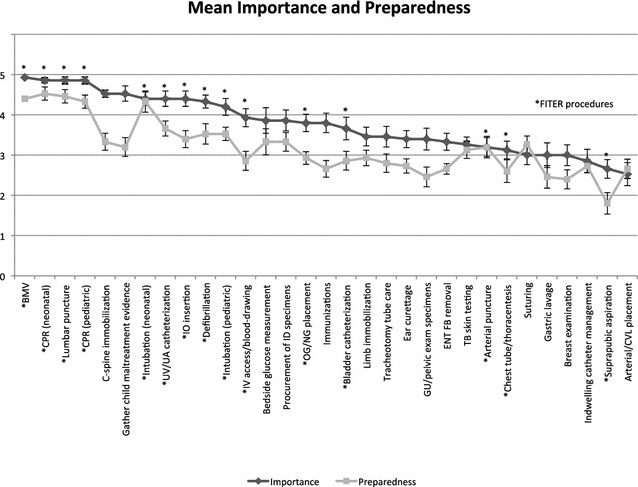
Mean perceived importance and preparedness of procedures. Mean perceived importance (*dark line*) and preparedness (*light line*) of the 31 procedural skills in order of most to least important. *Error bars* represent standard error. Procedures included in the FITER are indicated with an *asterisk*

We found a variation in the rated importance of the skills required of pediatric residents by the RCPSC. 12 skills had a mean importance rating greater than 4 (very or extremely important), 17 skills had a rating between 3 and 4 (important) and 3 had a rating <3 (somewhat or not important) (see Table 1). Those skills included in the FITER were spread out across the spectrum of perceived importance, and were not necessarily those felt to be most important by respondents. 11/26 (42 %) of procedures had a mean rating ≥4 (very important or extremely important), of which 9 were included in the FITER (Fig. 1). 7/26 (27 %) of procedures included in the FITER failed to achieve a mean rating of ≥4, including IV access/blood drawing, orogastric (OG)/nasogastric (NG) tube placement, bladder catheterization, arterial puncture, chest tube/thoracentesis, suturing/simple wound closure and suprapubic aspiration. Conversely, C spine immobilization and gathering child maltreatment evidence were skills felt to be important and not included in the FITER.

**Table 1:** Mean importance and preparedness

Most important			Moderately important			Least important		
Procedure	Importance	Preparedness	Procedure	Importance	Preparedness	Procedure	Importance	Preparedness
BMV^a^	4.93	4.4	IV access and blood-drawing^a^	3.93	2.86	Indwelling catheter management	2.85	2.73
CPR (neonatal)^a^	4.86	4.53	Bedside measurement of glucose	3.86	3.33	Suprapubic aspiration^a^	2.66	1.8
Lumbar puncture^a^	4.86	4.46	Procurement of ID specimens	3.86	3.33	Arterial/CVL placement	2.53	2.66
CPR (pediatric)^a^	4.86	4.33	OG/NG placement^a^	3.8	2.93			
C-spine immobilization	4.53	3.33	Immunizations	3.8	2.66			
Gather child maltreatment evidence	4.53	3.2	Bladder catheterization^a^	3.66	2.86			
Intubation (neonatal)^a^	4.4	4.33	Limb immobilization	3.46	2.93			
UV/UA catheterization^a^	4.4	3.66	Tracheotomy tube care	3.46	2.8			
IO insertion^a^	4.4	3.4	Ear curettage	3.4	2.73			
Defibrillation^a^	4.33	3.53	GU/pelvic exam/specimens	3.4	2.46			
Intubation (pediatric)^a^	4.2	3.53	ENT foreign body removal	3.33	2.66			
			TB skin testing	3.26	3.13			
			Arterial puncture^a^	3.2	3.2			
			Chest tube/thoracentesis^a^	3.13	2.6			
			Suturing^a^	3	3.26			
			Gastric lavage	3	2.46			
			Breast examination	3	2.4			

^a^Procedures included in the pediatrics FITER

Some procedures were felt to be relatively unimportant. 3/26 (12 %) procedures achieved a mean rating of <3 (somewhat or not important) (access/care for indwelling catheters, suprapubic aspiration and arterial/central venous line [CVL] placement). Those procedures included in the FITER were felt to be more important overall; mean importance of those procedures included in the FITER was 4.13 (SD 0.66), while those not included in the FITER was 3.43 (SD 0.58) (p < 0.0001).

### Preparedness

Similar to ratings of importance, ratings of preparedness were spread across the spectrum. Skills included in the FITER achieved a range of preparedness ratings. Residents were felt to be extremely or very well prepared (mean rating ≥4) for 5/26 (19 %) of procedures, all of which were included in the FITER (neonatal cardio-pulmonary resuscitation [CPR], lumbar puncture, bag-mask ventilation [BMV], pediatric CPR and neonatal tracheal intubation). They were found to be somewhat or not prepared (mean <3) for 15/26 (58 %) of procedures, 5/26 (19 %) of which were included in the FITER (OG/NG tube placement, IV access/blood drawing, bladder catheterization, chest tube placement/thoracentesis and suprapubic aspiration) (Fig. 1). Overall, procedures included in the FITER achieved greater perceived preparedness; mean perceived preparedness of procedures included in the FITER was 3.48 (SD 0.78), while those not included in the FITER was 2.91 (SD 0.33) (p < 0.0001).

**Fig. 2:**
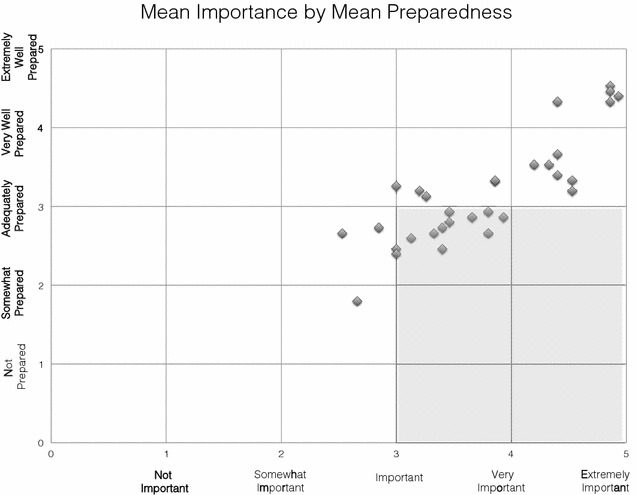
Mean perceived importance by mean perceived preparedness. *Scatter plot* of mean perceived importance (x axis) by mean perceived preparedness (y axis). *Gray* area shows skills that are perceived to be of high importance (>3) but low preparedness (<3): IV access/blood drawing, OG/NG tube placement, immunizations, bladder catheterization, limb immobilization, tracheostomy tube care, ear curettage, GU/pelvic exam/specimens, ENT foreign body removal, and chest tube/thoracentesis

Importance and preparedness for each procedure were compared. Ten procedures with a mean importance rating >3 had a mean preparedness rating of <3: IV access/blood drawing, OG/NG tube placement, immunizations, bladder catheterization, limb immobilization, tracheostomy tube care, ear curettage, GU/pelvic exam/specimens, ENT foreign body removal, and chest tube/thoracentesis (see Fig. 2). Four of these procedures are contained in the FITER (IV access/blood drawing, OG/NG tube placement, bladder catheterization, and chest tube/thoracentesis).

### Curriculum and documentation

**Fig. 3:**
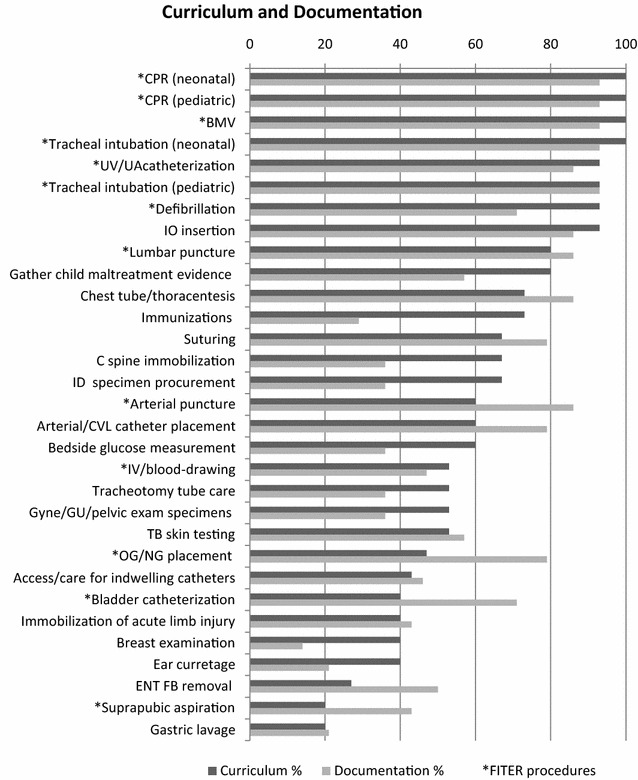
Curriculum and documentation of procedures. Percentage of respondents indicating presence of a curriculum (*dark line*) and documentation of procedures (*light line*). Procedures included in the FITER are indicated with an *asterisk*

Figure 3 depicts the number of respondents who indicated that they had a curriculum in place and that they documented competency in each procedure. Curriculum was more often present for procedures felt to be of greater importance with curriculum being strongly correlated with mean importance (r = 0.7, p < 0.0001). Residents were likewise perceived to achieve greater preparedness for those procedures with a curriculum in place, with presence of a formal curriculum being strongly correlated with mean perceived preparedness (r = 0.8, p < 0.0001). Documentation of skills was also strongly correlated with perceived importance (r = 0.7, p < 0.01) and moderately correlated with perceived preparedness (r = 0.5, p < 0.01). All correlations, except for documentation and perceived preparedness, which was moderate, were high and positive indicating strong relationships. The correlations were statistically significant, indicating that despite relatively low numbers (N = 16) these relationships did not occur by chance.

## Discussion

The SCP of the RCPSC is the standard setting body for post-graduate medical training in pediatrics in Canada. The SCP develops the OTR and FITER in pediatrics. Our study found that the pediatric procedural skills required by the RCPSC are felt to be of varying importance by residency PDs, and that residents’ are felt to graduate with a range of preparedness in these skills. Residents are perceived to achieve greater preparedness in those skills taught through formal residency curricula, and with some exceptions, the skills included in curricula are generally those that are felt to be most important. Overall skills included in the pediatric FITER are felt to be important, and residents achieve greater preparedness in these skills than in others.

The FITER is an important method of documenting and deeming a resident fit for independent practice in Canada. Our study shows that there are several skills in the pediatric FITER that are felt to be less important to practice (arterial puncture, chest tube placement, suprapubic aspiration). In contrast, there are skills that are not included in the FITER which are felt to be important to practice (C spine immobilization, gathering evidence of child maltreatment). Based on our study’s findings, this list of procedural skills warrants critical review and perhaps revisions. In the United States, the Pediatric Residency Review Committee requires training in only 16 procedures, and yet residents still struggle to achieve competency [2]. Refining the list of procedures required by the RCPSC and including those considered most important in the FITER may help focus training on those skills which are deemed to be most important, or where residents are currently failing to reach an adequate level of preparedness by the time of graduation. Focused training on specific procedures may help residents gather the volume of repeated, deliberate practice necessary to become competent in these particular procedures. As has been observed by Ericsson, significant improvements in individual performances were realized when individuals were given a task with a defined goal, motivated to improve, provided with feedback, and provided with ample opportunities for repetition and gradual refinements of their performance [19].

The gap between perceived importance and perceived preparedness is likewise important to examine. It is concerning that residents are not felt to be adequately prepared in several skills where importance is rated quite highly, namely IV access/blood drawing, OG/NG tube placement, immunizations, bladder catheterization, limb immobilization, tracheostomy tube care, ear curettage, GU/pelvic exam/specimens, ENT foreign body removal, and chest tube/thoracentesis. The presence of formal curricula for these skills was variable across programs, but generally high. The best way to teach these particular procedures may need to be examined, and these may be procedures for programs with limited resources to target for further curricular interventions.

The finding of a mismatch between perceived importance and preparedness is consistent with the study by Gaies, as well as others conducted in other fields including internal and family medicine [17, 18, 20–22]. In contrast to the study by Gaies et al., however, PDs in our study felt that procedures that are part of resuscitation skills (e.g. intubation, defibrillation) were almost universally important. In the Gaies study <75 % of PDs rated these procedures as very or extremely important, whereas in our study all resuscitation procedures were rated as very or extremely important. This may reflect a difference in US versus Canadian training programs; programs in the US tend to include more community based and primary care pediatrics, whereas those in Canada are based more in tertiary care pediatric centers, where resuscitation skills may be more frequently emphasized and utilized.

Relying on clinical exposure for residents to learn necessary skills may not be enough. We found that residents spend the majority of their time (77 %) in university or academic centers. In these settings, opportunities to learn procedural skills may be unevenly distributed [12]. The presence of multiple competing trainees (e.g. subspecialty fellows, respiratory therapy students, etc.) and other ancillary services (e.g. IV or phlebotomy teams) may dilute the potential for procedural experience. A recent study at our institution showed that residents perform very few procedures on the wards, spending more time on indirect patient care and paperwork [23]. This lack of procedural exposure leads to the need for specific curricula to teach and maintain procedural skills for our residents. Gaies showed that the use of any method to teach procedures formally was associated with increased perceived competence of residents to perform that procedure; similarly, our study shows that a formal curriculum is associated with improved perception of preparedness. However the relationship between these perceptions and true competence or preparedness remains questionable as residents very often overestimate their skills. Procedural training programs have been well described in other specialties [21, 24], and more recently in pediatrics [25]. Interestingly, we found that in Canadian residency training programs, the curriculum often did not focus on skills included in the FITER, nor on those procedures felt to be most important. Although they are clearly associated with improved perceived performance, the choice of procedures to include in formal training programs is not clear. Over the last decade, many programs have begun to use simulation as a teaching and evaluation mechanism for learning procedural skills. Simulation has focused primarily on resuscitation and acute care skills [26–28], often in subspecialty rotations [29]. The use of simulation to teach less acute skills has not been as well described and is less well developed. Simulation offers a chance for residents to attempt well-defined tasks with the appropriate level of difficulty and ample opportunity for feedback, repetition, and correction of errors [30]. A recent meta-analysis has in fact shown that simulation-based curricula with deliberate practice may be superior to traditional clinical medical education in achieving specific clinical skill acquisition goals [31]. Given the time constraints and practical limitations of traditional clinical exposure, a simulation curriculum may be appropriate to target skills noted in our study to have high perceived importance but low perceived preparedness ratings.

Our study has limitations. Responses from 2 of the 17 programs were not obtained, and we do not know whether responses from these programs may have differed from those that did respond. There were no differences found based on respondent characteristics, however the study was not powered to evaluate for these differences. This study was originally conducted in 2010, and several PD’s have changed since that time, making it possible that findings do not reflect the opinions of current PDs. However, given that the list of procedures has not changed, we feel the results are still relevant. Second, our study design did not include a negative control procedure (a procedure such as sigmoidoscopy that is not required by the RCPSC for training pediatricians). The inclusion of a negative control procedure may have helped establish a reference point for readers to interpret the results and ensure that participants truly understood the questions. Third, the results are the perceptions of PDs, and not necessarily the actual importance or preparedness of graduating residents. Furthermore, opinions on preparedness for training often differ between residents and their supervisors [1]. Ideally, though difficult to study, documentation of actual levels of preparedness in real life are necessary to fully understand procedural training in pediatric residency. Future studies in this area should focus on the opinions of trainees as well as practicing pediatricians to further elucidate the relative importance and preparedness of the list of required skills. However, while opinions of PDs may not be generalizable, their opinions are vitally important. PDs contribute to curricula development and may determine a program’s emphasis on the various aspects of training and so have a great deal of power over resident education. The importance of their opinions should not be underestimated.

## Conclusions

Many but not all procedures in which pediatric residents are required to be competent by the RCPSC are felt to be important, and procedures included in the FITER are not necessarily those felt to be most important. Residents are not felt to be adequately prepared in several of the required procedures. Formal procedural curricula are helpful, and procedures with high importance but low preparedness ratings should be targeted for curricular interventions. Simulation is a modality that could be further explored for these particular skills (Additional file 1).

## Additional file

10.1186/s13104-015-1499-8 Pediatric procedural skills survey.

## Abbreviations

BMV: bag mask ventilation
ITER: in-training evaluation report
CPR: cardio-pulmonary resuscitation
CVL: central venous line
FITER: final in-training evaluation report
IV: intravenous
NG: naso-gastric
OG: oro-gastric
OSCE: objective structured clinical exam
OTR: objectives of training
PD: program director
RCPSC: Royal College of Physicians and Surgeons of Canada
SCP: Specialty Committee in Pediatrics
