# Supplementary material for: Program directors’ perceptions of importance of pediatric procedural skills and resident preparedness
Source: BMC Res Notes. 2015 Oct 9;8:550. doi: 10.1186/s13104-015-1499-8 (PMC4600326; doi:10.1186/s13104-015-1499-8)
Supplement: Supplementary file 1 — 10.1186/s13104-015-1499-8 Pediatric procedural skills survey. [file 13104_2015_1499_MOESM1_ESM.pdf]

Pediatric Procedural Skills Survey

1. Are you a Program Director or 'nucleus' member of the Pediatric Specialty Committee?

○ Program Director
○ Nucleus Pediatric Specialty Committee Member

2. Year of completion of General Pediatric residency training ___________

3. Primary specialty

○ Adolescent medicine
○ Cardiology
○ Clinical Allergy/Immunology
○ Critical Care Medicine
○ Dermatology
○ Developmental Pediatrics
○ Emergency Medicine
○ Endocrinology and Metabolism
○ Gastroenterology
General Pediatrics (check below)
  ○ Academic General Pediatrics
  ○ Consulting General Pediatrics
○ Community Pediatrician
○ Hospital Pediatrics (Hospitalist)
○ Genetics/Metabolism
○ Hematology/Oncology
○ Infectious Diseases
○ Neonatal-Perinatal Medicine
○ Nephrology
○ Neurology
○ Respirology
○ Rheumatology

○Other _______

4. Total number of pediatric residents in your program ________

5. What percentage of time do your residents spend in the following settings?

A. University hospital or university setting (e.g., CHWO, MUMC) ______%
B. Community ambulatory settings ______%
C. Community hospital(s) ______%

6. In the past 5 years, what percentage of your residents have pursued subspecialty pediatric training after completion of their core pediatric training? ______%

7. In the past 5 years, what percentage of your residents have pursued subspecialty fellowship training in Cardiology, Critical Care, Emergency Medicine, and Neonatology? ______%

8. Is there a phlebotomy team that draws blood samples at your institution(s)?
○Yes – Daytime only
○Yes – 24-hours
○No

9. Do you have an IV-start team at your institution(s)?
○Yes – Daytime only
○Yes – 24-hours
○No

10. How often do residents do the following?

	Never<br>1	Infrequently<br>2	Sometimes<br>3	Frequently<br>4	Very Frequently<br>5
**Draw blood samples**					
**Start IV's**					

11. For the purpose of this survey, competency is defined as the ability to perform a procedure or skill independently, without supervision, and with a high likelihood of successful completion. This includes an understanding of the indications, contraindications, and risks of the procedure or skill.

In your opinion, how important is the competency of performing the following:

	Not important	Somewhat important	Important	Very important	Extremely important
	1	2	3	4	5
Intravenous access and blood-drawing					
Umbilical venous and umbilical arterial catheterization					
Arterial puncture					
Arterial line/central venous catheter placement					

continued	*Not important*	*Somewhat important*	*Important*	*Very important*	*Extremely important*
	1	*2*	*3*	*4*	*5*
Suture of a one layer laceration, simple wound closure					
Cardiopulmonary resuscitation (neonatal)					
Cardiopulmonary resuscitation (pediatric)					
Bag-mask ventilation					
Tracheal intubation (neonatal)					
Tracheal intubation (pediatric)					
Defibrillation					
Lumbar puncture					
Bladder catheterization					
Suprapubic aspiration					
Gastric tube placement (oro or nasogastric)					
Gastric lavage					
Intraosseous insertion					
Chest tube placement and thoracentesis					
Access and care for indwelling catheters					
Perform and interpret oximetry					
Tracheotomy tube care, including replacement					
Foreign body removal from eye/nose/upper airway					
Immobilization of acute limb injury including fractures					
Cervical spine immobilization					
Gynecological, genito-urinary and pelvic examination and specimen procurement					
Breast examination					
Reliably interpret an electrocardiogram in all age groups					
Interpret a chest X-ray with respect to heart size, contour and pulmonary vascularity					
Bedside measurement of glucose					
Interpretation of abdominal X-rays					
Tuberculin skin testing - perform and interpret					
Procurement of appropriate specimens for diagnosis of infections					
Immunizations (storage, administration and documentation)					

continued	*Not important*	*Somewhat important*	*Important*	*Very important*	*Extremely important*
	1	*2*	*3*	*4*	*5*
Interpret bone X-rays for fractures					
Perform curettage under direct visualization of the ear					
Interpretation of the tympanogram					
Interpretation of soft tissue X-rays in acute upper airway obstruction					
Interpret common abnormalities seen on urine microscopy					
Interpretation of pulmonary function tests					
Gather child maltreatment evidence appropriately including documentation and specimen collection					

13. In your opinion, how well prepared are residents in your program to perform the following by the end of their residency?

	**Not prepared**	**Somewhat prepared**	**Adequately prepared**	**Very well prepared**	**Extremely well prepared**
	**1**	**2**	**3**	**4**	**5**
Intravenous access and blood-drawing					
Umbilical venous and umbilical arterial catheterization					
Arterial puncture					
Arterial line/central venous catheter placement					
Suture of a one layer laceration, simple wound closure					
Cardiopulmonary resuscitation (neonatal)					
Cardiopulmonary resuscitation (pediatric)					
Bag-mask ventilation					
Tracheal intubation (neonatal)					
Tracheal intubation (pediatric)					
Defibrillation					
Lumbar puncture					
Bladder catheterization					
Suprapubic aspiration					

continued	*Not prepared*	*Somewhat prepared*	*Adequately prepared*	*Very well prepared*	*Extremely well prepared*
	1	*2*	*3*	*4*	*5*
Gastric tube placement (oro or nasogastric)					
Gastric lavage					
Intraosseous insertion					
Chest tube placement and thoracentesis					
Access and care for indwelling catheters					
Perform and interpret oximetry					
Tracheotomy tube care, including replacement					
Foreign body removal from eye/nose/upper airway					
Immobilization of acute limb injury including fractures					
Cervical spine immobilization					
Gynecological, genito-urinary and pelvic examination and specimen procurement					
Breast examination					
Reliably interpret an electrocardiogram in all age groups					
Interpret a chest X-ray with respect to heart size, contour and pulmonary vascularity					
Bedside measurement of glucose					
Interpretation of abdominal X-rays					
Tuberculin skin testing - perform and interpret					
Procurement of appropriate specimens for diagnosis of infections					
Immunizations (storage, administration and documentation)					
Interpret bone X-rays for fractures					
Perform curettage under direct visualization of the ear Interpretation of the tympanogram					
Interpretation of soft tissue X-rays in acute upper airway obstruction					
Interpret common abnormalities seen on urine microscopy					

continued	*Not prepared*	*Somewhat prepared*	*Adequately prepared*	*Very well prepared*	*Extremely well prepared*
	1	*2*	*3*	*4*	*5*
Interpretation of pulmonary function tests					
Gather child maltreatment evidence appropriately including documentation and specimen collection					

14. For the purpose of the following questions, a curriculum can be defined as any of the following: didactic sessions, formal observation of procedures performed by experts, or observed practice/simulation.

	**Do you have a standardized curriculum for teaching this procedure or skill?**
Intravenous access and blood-drawing	○ Yes ○ No
Umbilical venous and umbilical arterial catheterization	○ Yes ○ No
Arterial puncture	○ Yes ○ No
Arterial line/central venous catheter placement	○ Yes ○ No
Suture of a one layer laceration, simple wound closure	○ Yes ○ No
Cardiopulmonary resuscitation (neonatal)	○ Yes ○ No
Cardiopulmonary resuscitation (pediatric)	○ Yes ○ No
Bag-mask ventilation	○ Yes ○ No
Tracheal intubation (neonatal)	○ Yes ○ No
Tracheal intubation (pediatric)	○ Yes ○ No
Defibrillation	○ Yes ○ No
Lumbar puncture	○ Yes ○ No
Bladder catheterization	○ Yes ○ No
Suprapubic aspiration	○ Yes ○ No
Gastric tube placement (oro or nasogastric)	○ Yes ○ No
Gastric lavage	○ Yes ○ No
Intraosseous insertion	○ Yes ○ No
Chest tube placement and thoracentesis	○ Yes ○ No
Access and care for indwelling catheters	○ Yes ○ No
Perform and interpret oximetry	○ Yes ○ No
Tracheotomy tube care, including replacement	○ Yes ○ No
Foreign body removal from eye/nose/upper airway	○ Yes ○ No
Immobilization of acute limb injury including fractures	○ Yes ○ No
Cervical spine immobilization	○ Yes ○ No
Gynecological, genito-urinary and pelvic examination and specimen procurement	○ Yes ○ No

continued	*Do you have a standardized curriculum for teaching this procedure or skill?*	
Breast examination	○ Yes	○ No
Reliably interpret an electrocardiogram in all age groups	○ Yes	○ No
Interpret a chest X-ray with respect to heart size, contour and pulmonary vascularity	○ Yes	○ No
Bedside measurement of glucose	○ Yes	○ No
Interpretation of abdominal X-rays	○ Yes	○ No
Tuberculin skin testing - perform and interpret	○ Yes	○ No
Procurement of appropriate specimens for diagnosis of infections	○ Yes	○ No
Immunizations (storage, administration and documentation)	○ Yes	○ No
Interpret bone X-rays for fractures	○ Yes	○ No
Perform curettage under direct visualization of the ear	○ Yes	○ No
Interpretation of the tympanogram	○ Yes	○ No
Interpretation of soft tissue X-rays in acute upper airway obstruction	○ Yes	○ No
Interpret common abnormalities seen on urine microscopy	○ Yes	○ No
Interpretation of pulmonary function tests	○ Yes	○ No
Gather child maltreatment evidence appropriately including documentation and specimen collection	○ Yes	○ No

15. For the purpose of the following questions, documentation may include procedure logs, evaluation using simulations, OSCE evaluation or other methods resulting in a formal record of competence.

	**Do you document the competence of your residents to perform this procedure or skill?**	
Intravenous access and blood-drawing	○ Yes	○ No
Umbilical venous and umbilical arterial catheterization	○ Yes	○ No
Arterial puncture	○ Yes	○ No
Arterial line/central venous catheter placement	○ Yes	○ No
Suture of a one layer laceration, simple wound closure	○ Yes	○ No
Cardiopulmonary resuscitation (neonatal)	○ Yes	○ No
Cardiopulmonary resuscitation (pediatric)	○ Yes	○ No
Bag-mask ventilation	○ Yes	○ No
Tracheal intubation (neonatal)	○ Yes	○ No
Tracheal intubation (pediatric)	○ Yes	○ No

continued	*Do you document the competence of your residents to perform this procedure or skill?*	
Defibrillation	○ Yes	○ No
Lumbar puncture	○ Yes	○ No
Bladder catheterization	○ Yes	○ No
Suprapubic aspiration	○ Yes	○ No
Gastric tube placement (oro or nasogastric)	○ Yes	○ No
Gastric lavage	○ Yes	○ No
Intraosseous insertion	○ Yes	○ No
Chest tube placement and thoracentesis	○ Yes	○ No
Access and care for indwelling catheters	○ Yes	○ No
Perform and interpret oximetry	○ Yes	○ No
Tracheotomy tube care, including replacement	○ Yes	○ No
Foreign body removal from eye/nose/upper airway	○ Yes	○ No
Immobilization of acute limb injury including fractures	○ Yes	○ No
Cervical spine immobilization	○ Yes	○ No
Gynecological, genito-urinary and pelvic examination and specimen procurement	○ Yes	○ No
Breast examination	○ Yes	○ No
Reliably interpret an electrocardiogram in all age groups	○ Yes	○ No
Interpret a chest X-ray with respect to heart size, contour and pulmonary vascularity	○ Yes	○ No
Bedside measurement of glucose	○ Yes	○ No
Interpretation of abdominal X-rays	○ Yes	○ No
Tuberculin skin testing - perform and interpret	○ Yes	○ No
Procurement of appropriate specimens for diagnosis of infections	○ Yes	○ No
Immunizations (storage, administration and documentation)	○ Yes	○ No
Interpret bone X-rays for fractures	○ Yes	○ No
Perform curettage under direct visualization of the ear	○ Yes	○ No
Interpretation of the tympanogram	○ Yes	○ No
Interpretation of soft tissue X-rays in acute upper airway obstruction	○ Yes	○ No
Interpret common abnormalities seen on urine microscopy	○ Yes	○ No
Interpretation of pulmonary function tests	○ Yes	○ No
Gather child maltreatment evidence appropriately including documentation and specimen collection	○ Yes	○ No

Thank-you for completing the survey!

Please add any comments or questions below.

Further questions or comments may be directed to:

Zia Bismilla, MD, M.Ed
zia.bismilla@sickkids.ca

Harish Amin, FRCPC
hamin@ucalgary
